# Anti-Bacterial Effect and Cytotoxicity Assessment of Lipid 430 Isolated from *Algibacter* sp.

**DOI:** 10.3390/molecules24213991

**Published:** 2019-11-05

**Authors:** Yannik K.-H. Schneider, Kine Ø. Hansen, Johan Isaksson, Sara Ullsten, Espen H. Hansen, Jeanette Hammer Andersen

**Affiliations:** 1 Marbio, Faculty for Fisheries, Biosciences and Economy, UiT—The Arctic University of Norway, Breivika, N-9037 Tromsø, Norwaysara.m.ullsten-wahlund@uit.no (S.U.); espen.hansen@uit.no (E.H.H.); jeanette.h.andersen@uit.no (J.H.A.); 2 Department of Chemistry, Faculty of Natural Sciences, UiT—The Arctic University of Norway, Breivika, N-9037 Tromsø, Norway; johan.isaksson@uit.no

**Keywords:** flavolipin, marine bacteria, natural products, lipopeptides, *algibacter*

## Abstract

Two bacterial isolates from the Barents Sea, both belonging to the genus *Algibacter*, were found to yield extracts with anti-bacterial bioactivity. Mass spectrometry guided dereplication and purification of the active extracts lead to the isolation of the same active principle in both extracts. The structure of the bioactive compound was identified via mass spectrometry and nuclear resonance spectroscopy and it turned out to be the known lipopeptide Lipid 430. We discovered and determined its previously unknown anti-bacterial activity against *Streptococcus agalactiae* and revealed a cytotoxic effect against the A2058 human melanoma cell line at significantly lower concentrations compared to its anti-bacterial concentration. Flow cytometry and microscopy investigations of the cytotoxicity against the melanoma cell line indicated that Lipid 430 did not cause immediate cell lysis. The experiments with melanoma cells suggest that the compound functions trough more complex pathways than acting as a simple detergent.

## 1. Introduction

**Figure 1:**
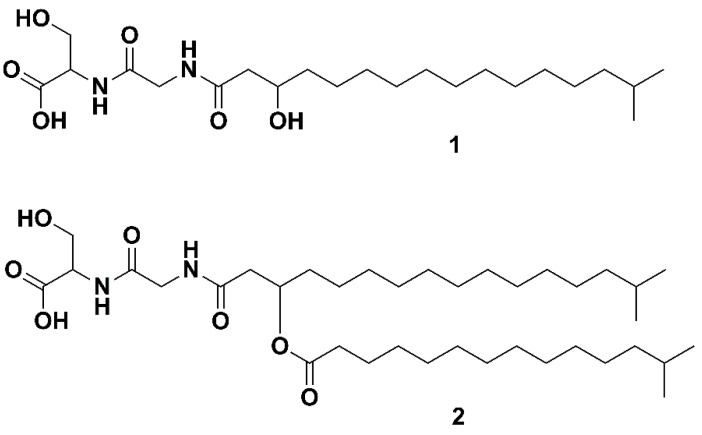
The two serine dipeptide lipids Lipid 430 (**1**) and Lipid 654 (**2**), according to [16].

The genus Bacteriocides represents the second most abundant bacterial phylum within the marine heterotrophic picoplankton [1]. Bacteriocides, to which *Flavobacteria* belong, have the enzymes required to degrade proteins and carbohydrates [2], and play an important role in the degradation of organic matter within the marine environment. Remarkably, the observation of the abundance of marine *Flavobacteria* and the hypothesis that their presence is linked to their ability to degrade algal polymers dates back to 1946 [3]. Within the *Flavobacteriaceae* family, the genus *Algibacter* was erected in 2004. It represents a taxon of rod-shaped, facultative anaerobic, Gram negative bacteria, unable to form endospores [4]. Its first representative, *Algibacter lectus,* was isolated from green algae and described in 2004 by Nedashkovskaya et al. [4]. Further representatives have been isolated from seawater [5,6], invertebrates [7] and from algae or in close proximity to them [4,8,9,10]. *Algibacter alginolytica* was isolated from a brown seaweed (*Laminaria japonica*). Sequencing and genomic analysis revealed that it has the highest proportion of carbohydrate-active enzymes (~7.5%) among the Flavobacteria. The bacterium was shown to hydrolyze Tween 20, Tween 40, Tween 60, Tween 80, galantine, alginate and starch, which indicates the ecological significance of *Algibacter* in breaking down algal biopolymers [10]. As part of this work, a lipopeptide known as Lipid 430 (**1**, Figure 1) was isolated. A novel serine dipeptide lipid, Lipid 654 (**2**, Flavolipin, Figure 1), was first isolated from *Flavobacterium menigosepticum* in 1988 [11,12]. *F. menigosepticum* is an opportunistic pathogen able to cause neonatal meningitis and nosocomial infections in immunocompromised individuals [13]. Stereo-controlled synthesis of **2** and bioactivity testing revealed that its observed macrophage activating effect [14] is triggered stereospecifically by the l-serine dipeptide lipid *N*-[*N*-[(3*R*)-15-methyl-3-(13-methyltetradecanoyloxy)hexadecanoyl]glycyl]-L-serine, showing the same bioactivity as natural **2** [12]. An early investigation suggested that **2** was a Toll-like receptor 4 ligand [15], but recent investigations have shown that it acts as ligand on human and murine Toll-like receptor 2 (TLR-2) [16]. Compound **1** (Figure 1) is also shown to trigger TLR-2 [16]. The structures of both lipids (Figure 1) have been verified by total synthesis [17]. In the previously mentioned study, **1** and **2** were isolated from *Porphyromonas gingivalis*. The pathogen is reported to be a virulence factor in destructive periodontal disease, and lipids, such as dihydroceramides, have been shown to be involved in TLR-2 mediated inflammation and inhibition of osteoblast differentiation [18,19]. Compounds **1** and **2** also inhibit osteoblast differentiation and function. Notably, the effect of **2** is mediated trough TLR-2 while the effect of **1** on osteoblasts is only partly mediated via TLR-2, indicating another target for **1** in osteoblasts beside TLR-2 [20]. Due to that, serine-dipeptide lipids, together with sphingolipids, are suggested to be virulence factors of *P. ginvialis* [21]. Interestingly, all those lipids likely to be virulence factors share the attribute of an isobranched aliphatic fatty acid as a common feature, but they have a large degree of variation in the head groups [21]. It has been shown that **1** and **2** are produced by commensal oral and intestinal bacteria of the Bacteroidetes phylum, and they can be detected in human tissue samples [22]. Notably, **2** is stereospecifically deacetylated by phospholipase 2, yielding a free fatty acid and **1** [17,23].

In the present study, we investigated two marine *Algibacter* sp. isolates for anti-microbial and anti-cancer activities. Dereplication of the bioactive extracts revealed that both contained the same unidentified compound and the purification of the compound led to the isolation and identification of the lipopeptide **1** from both *Algibacter* strains. The previously unknown anti-bacterial effect against *S. agalactiae* was investigated and its cytotoxicity against lung fibroblasts and two cancer cell lines was assessed.

## 2. Results

### 2.1. High Troughput Screening and Identification of the Strains

Through an in house high-throughput screening campaign where marine microorganisms were cultivated, extracted, fractionated and screened for potential anti-cancer and anti-microbial activities, two strains showed anti-microbial activity. Sequencing of the 16S rRNA and nucleotide blast against 16S ribosomal RNA sequences revealed that the strains M09B557 and M09B045 belong to the genus *Algibacter* (Sequences in Appendix A). Strain M09B557 was isolated from the bryozoan *Alcyonidium gelatinosum* and strain M09B045 was isolated from a soft coral commonly called “sea strawberry” (*Gersemia rubiformis*), both sampled in the Barents Sea.

### 2.2. Bioactivity Screening and Dereplication

**Figure 2:**
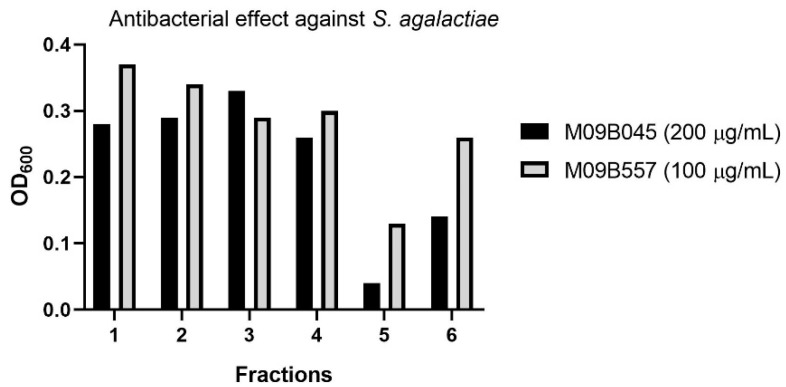
Anti-bacterial effect of the fractions generated by flash liquid chromatography from extracts of the cultures of the two *Algibacter* strains M09B045 and M09B557. Note that the tested assay concentrations are different for the two strains but the purpose of the test was to identify candidates for isolation rather than quantitative comparison of bioactivity.

The strains were recultivated in three 300 mL cultures each to produce sufficient material for confirming the bioactivity detected in the previous high throughput screening campaign. The raw extracts were fractionated into six fractions using reversed phase flash liquid chromatography and subsequently tested in cell-based anti-microbial and anti-cancer assays. At concentrations of 200 and 100 µg/mL, respectively, an anti-bacterial effect of fraction five from both extracts against *Streptococcus agalactiae* was detected (see Figure 2), while no cytotoxic effect was observed against A2058 melanoma cells at a concentration of 200 µg/mL. The six flash fractions from each extract were analyzed using UHPLC-HR-MS, and the active fractions five were compared with the “flanking” inactive fractions four and six in an attempt to identify the component(s) responsible for the observed bioactivity. By comparing the MS data of the active fraction with the flanking fractions it is possible to identify compounds that are only present in the active fraction or there in the highest abundance. In addition, extracts of the growth media were prepared according to the same protocol as used for bacterial cultures. The media references were fractionated and analyzed via UHPLC-HR-MS to be compared to the extracts in order to exclude media-components present within the bacterial extracts. Compounds that were unique to the active fraction or present in higher amounts than in the inactive fractions, were further investigated by calculation of elemental compositions, and along with the MS-fragments, they were used for database searches. Using this approach, we were able to identify a candidate with a positive ion mass of *m*/*z* 431.3103 and retention time of 8.28 min present in fraction five from the extracts of both M09B557 and M09B045.

### 2.3. Isolation of Lipid 430

For isolation of **1**, 16 × 450 mL of M09B557 and 12 × 450 mL of M09B045 were cultivated, extracted and fractionated. The resulting flash fraction five from each extract was pooled, dried and dissolved in DMSO (40 mg/mL) and then diluted 1:4 (*v*/*v*) in methanol. For the first HPLC-purification step, a column with C-18 functionalized stationary phase was employed. Different gradients were used to purify the target compound with fraction collection triggered by retention time. The collected fractions were reduced to dryness by vacuum centrifugation and redissolved in methanol. For the second HPLC-purification step a fluorophenyl column in combination with mass guided fraction triggering was chosen. The final yields of the isolated compound were 1.7 mg from M09B045 and 2.3 mg from M09B557. The purities of the preparations were tested using UHPLC-HR-MS and the two samples were pooled. The chromatograms (BPI and extracted ion chromatogram as well as A_254nm_) of the purity test are given in the Supplementary Information (Figure S1).

### 2.4. Structure Elucidation via NMR and MS/MS Analysis

**Figure 3:**
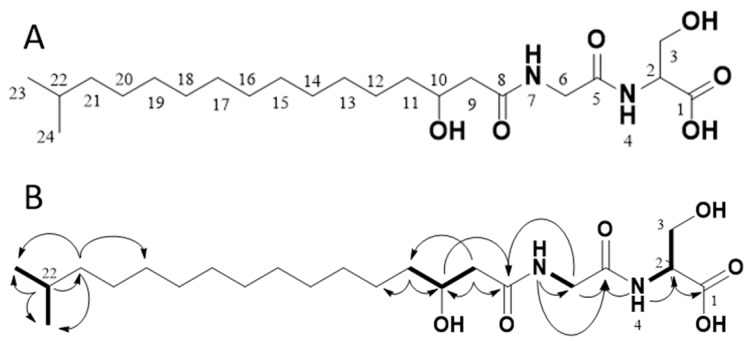
The structure of the isolated compound Lipid 430 (**1**) (**A**) and 2D-NMR correlations measured from our isolated sample (**B**). In B, selected COSY correlations are indicated in bold bonds and selected HMBC correlations are shown as arrows. The structure proposed upon the NMR data complies with Lipid 430 (**1**).

Through 1D (^1^H, ^13^C, Table 1) and 2D (HMBC, HSQC, H2BC, COSY, Figure 3 and Figures S2–S6 in the Supplementary Information) data recorded for **1**, the compound was confirmed to be Lipid 430. Due to significant overlap of the central CH_2_ groups (13-CH_2_ to 19-CH_2_), in agreement with what has previously been observed for **1** as well as the ester-linked iso C15:0 variant of **1** [16], these methylene groups could not be unambiguously assigned by NMR, though the integral sum of the unresolved region was consistent with the expected number of contributing protons. Based on HR-MS/MS the elemental composition was calculated to be C_22_H_42_N_2_O_6_ (*m*/*z* 431.3112 [M + H]^+^ in ESI+, calcd 431.3121 and 429.2970 [M − H]^−^ in ESI−, calcd 429.2965). Taking the MS results and the NMR spectra together, the proposed structure is the only conformation that fits both datasets.

### 2.5. Anti-microbial effect of Lipid 430

**Figure 4:**
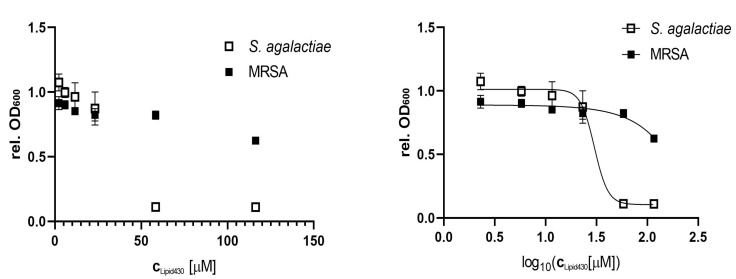
Anti-microbial effect of Lipid 430 (**1**) on *S. agalactiae* (two technical replicates in two experiments) and MRSA (two technical replicates). IC_50_ for *S. agalactiae* was 30.16 µM using a sigmoidal fit (Span ± 0.91 µM; Degrees of Freedom 20; R squared 0.97; Adjusted R squared 0.97, Sum of squares 0.13). IC_50_ of MRSA is >116 µM and was not determined.

Compound **1** was tested for anti-bacterial activity against *S. aureus*, *E. coli*, *E. faecialis*, *P. aeruginosa*, *S. agalactiae* and Methicillin resistant *S. aureus* (MRSA) at concentrations of 50, 25, 10, 5, 2.5 and 1 µg/mL, equal to molar concentrations of 116, 58, 23, 12, 6 and 2 µM, respectively. The tests were conducted twice, using two technical replicates in two independent experiments for *S. agalactiae*. A significant effect on *S. agalactiae* and MRSA was observed, see Figure 4. The calculated IC_50_ of **1** against *S. agalactiae* was 30 µM or 13 µg/mL respectively. At a concentration of 58 µM the growth of *S. agalactiae* was completely inhibited. For MRSA the IC_50_ was not determined as the highest tested concentration of (**1**) (116 µM or 50 µg/mL) reduced growth by 38%. To test if the observed effect on *S. agalactiae* was bactericidal or bacteriostatic, the 100 µL incubation volume of the growth assay for 58 µM **1** was streaked out further on brown agar and incubated at 37 °C. No colony or sign of bacterial growth was visible after 4 days of incubation (two technical replicates). There were no colonies formed after incubation with the compound, which indicated that **1** exerted bactericidal effect against *S. agalactiae* at a concentration of 58 µM.

### 2.6. Cytotoxic Effect of **1**

#### 2.6.1. Cytotoxicity Assay

**Figure 5:**
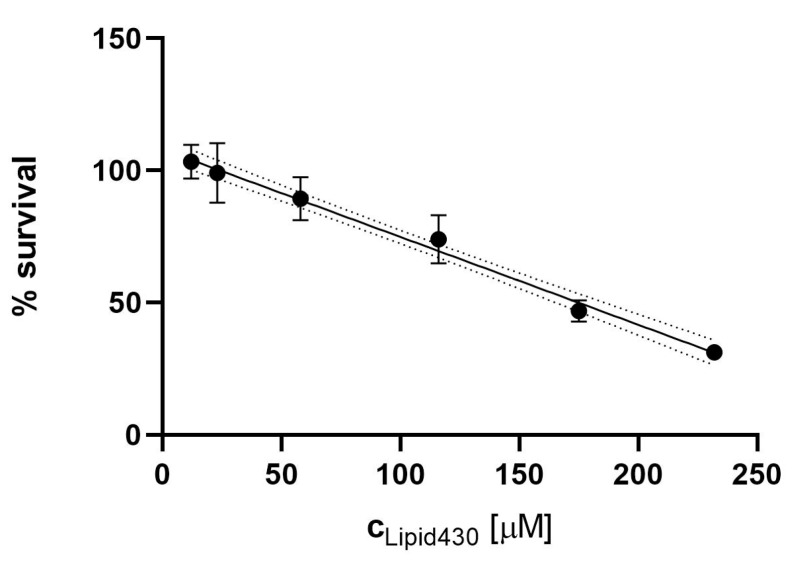
Cytotoxic effect of Lipid 430 (**1**) against the A2058 melanoma cell line. A linear correlation with % Survival = −0.00332c+1.08 {c ∈ ℝ | c ≥ 12 µM ^ c ≤ 233 µM} was found (R square 0.93, Sy.x 0.07442). The 95% Confidence intervals are shown in dot lines. The calculated IC_50_ for **1** in the linear model is 175 µM.

The effect of **1** was tested against three human cell lines, the melanoma cell line A2058, the colon carcinoma cell line HT29 and the lung fibroblast cell line MRC5. The compound was tested at concentrations of 100, 75, 50, 25, 10 and 5 µg/mL equal to molar concentrations of 233, 175, 116, 58, 23 and 12 µM, respectively. There was no significant effect observed against the lung fibroblast or colon carcinoma cells at the tested concentrations. The results for all tested cell lines and positive controls are shown in the Supplementary Information (Figure S7). For the melanoma cell line, a dose dependent cytotoxic effect was observed, see Figure 5. The IC_50_ of **1** against the melanoma cell line was calculated to be 175 µM (75 µg/mL). The test was executed in two independent experiments with three technical replicates each.

#### 2.6.2. Propidium Iodide Staining and Flow Cytometry

**Figure 6:**
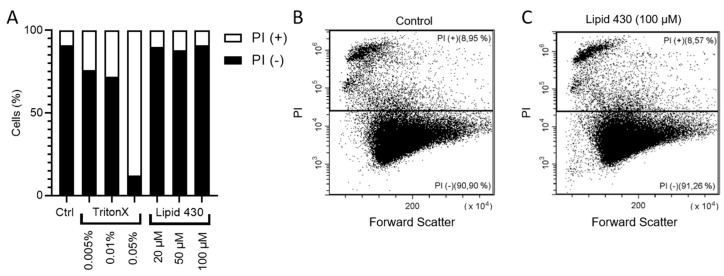
Results of the flow cytometry experiments with melanoma cell line A2058. In (**A**) the relative ratio between PI positive (+) and PI negative (−) is shown. The exact results are the following: stained control (Ctrl.), 8.46% PI+; 0.01% TritonX, 27.61% PI+; 0.05% TritonX, 87.38% PI+; 20 µM **1**, 9.45% PI+; 50 µM Lipid 430, 11.26% PI+; 100 µM Lipid 430, 8.72% PI+. (**B**) depicts the flow cytometry results as dot plot graph of the stained control and in (**C**) a dot plot graph of the cells threated with 100 µM **1** is given. Forward scatter is displayed on the X-axis and propidium iodide absorption on the Y-axis. The relative ratio of events is given in %.

To investigate whether the cytotoxic effect of **1** was mediated by affecting the integrity of the cell membrane, propidium iodide (PI) staining in combination with flow cytometry was employed. PI is indicating integrity of the cell membrane by passing through damaged membranes and intercalating into the DNA. As a positive control, TritonX™ was tested at concentrations of 0.005, 0.01 and 0.05%. (*v*/*v*). Compound **1** was tested at concentrations of 20, 50 and 100 µM. The results are shown in Figure 6. The PI positive cells indicate the population of cells with affected cell membranes increasing with the concentration of the detergent TritonX. For **1**, no tendency was observable (see Figure 6A). The exemplary dot plot graphs of the Control and of 100 µM **1** support this assumption (see Figure 6B,C). Due to the limited amount of compound, the experiment was carried out only once, this should be considered critically when interpreting the gained data. The dot plot graphs for all conditions are given in the Supplementary Information S8.

#### 2.6.3. Microscopic Investigation of the Melanoma Cell Line A2058

For the microscopic examination, the cells were exposed for 4 h to concentrations of 100- and 500 µg/mL of **1**, equal to molar concentrations of 233 µM and 1165 µM, respectively. In both cases, no morphological difference between the treatment and the control could be observed at 100× magnification. Microscopic pictures of the investigation are shown in the Supplementary Information (Figure S9).

### 2.7. Lipid Isolation, Detection of Lipid 654

Compound **1** is known to be a de-acetylation product of **2** catalyzed by phospholipases. Likewise, **1** could be the biosynthetic predecessor of **2** trough esterification of **1**. Therefore, the raw extracts of both bacteria were analyzed using UHPLC-HR-MS in order to look for **2**, but no mass signal was found that could be related to **2**. To ensure that the absence of **1** was not a result of the extraction protocol using HP-20 beads, chloroform extraction was executed with cultures of both strains. The chloroform extracts were analyzed using UHPLC-HR-MS, but no signals potentially related to **2** were detected.

## 3. Discussion

The active principle of the two extracts from two *Algibacter* isolates was identified and investigated upon its bioactivity towards bacteria and mammalian cell lines. The observed anti-bacterial effect of **1** against *Streptococcus agalactiae* was higher compared to the pathogen MRSA which possessed a significantly higher tolerance against **1**. Notably, *S. agalactiae* was the most sensitive among the tested bacterial strains. When screening the bacterial extracts, we frequently observed that fractions containing for instance phosphocolines or rhamnolipids were active against *S. agalactiae* while no or only weak activities were observed against the other bacteria (data not shown) [24]. The sensitivity of the melanoma cell line against **1** was significantly (at least seven times) lower compared to the anti-bacterial effect against *S. agalactiae*. Furthermore, no effects were observed on colon carcinoma cells and lung fibroblasts. This corresponds well with the observation that the initial screening of the flash fractions of the crude extracts did not show activity in the anti-cancer assays while it did in the anti-microbial assays.

The fact that **1** showed activity against the bacterial strain and cancer cell line that in our experience are most sensitive to surfactants gives rise to the suspicion that the compound is affecting the integrity of the cell membranes in an unspecific way. Given the known bioactivity of **1**, being a ligand to TLR-2 on one hand and the structure of the molecule on the other one, it was questionable if the cytotoxic effect was mediated by lysing the cells. The aliphatic, iso-branced fatty acid with a polar head consisting of two amino acids could suggest that it acts as surfactant. Therefore, PI staining followed by flow cytometry analysis was done to check if the lipid affected the membrane integrity of melanoma cells. This turned out to not be the case for any of the tested concentrations. PI staining is a technique capable of staining cells with reduced membrane integrity that can be detected by flow cytometry [25,26] with high linearity [27]. We used TritonX™ as detergent to test the suitability of the method. However, we did not observe a cellular effect after one hour of incubation with propidium iodide when analyzed with flow cytometry or after 4 h when inspecting the cells in the microscope, at least not at the tested concentrations. The effect we detected in the cytotoxicity assay was observed after 72 h of incubation with **1**, conclusively the effect is taking place during a longer incubation time maybe affecting cell division or cell cycle.

It is known that lipopeptides have a broad spectrum of activity including anti-fungal, anti-bacterial, anti-cancer and anti-inflammatory effects [28,29,30]. The lipopeptide antibiotic daptomycin is used to treat infections by Gram positive bacteria and was introduced into the marked in 2001 [31]. Surfactin, a lipopeptide with high surfactant power [32], exhibits also various bioactivities such as anti-inflammatory, anti-cancer and thrombolytic bioactivities [30]. The anti-bacterial and cytotoxic mode of action of both compounds relies on affecting the integrity of the cell membrane of target cells [30,33]. However, those two marketed lipopeptides differ significantly from **1**. Daptomycin is a 13 amino acid cyclic lipopeptide (10 amino acids forming a ring structure) linked to decanoic acid [34] while Surfactin consists of a seven aminoacid cycle, linked to a 13−15 carbon chain [35]. Note that there are also linear lipopeptides as for example gageostatins, isolated from a marine *Bacillus*, showing similar bioactivities [36]. However, the mentioned cyclic lipopeptides differ greatly from the linear two-amino acid **1** in structure and molecular mass. Even the Gageostratins with an Mw > 1000 u appear to be rather distant relatives. It seems more appropriate to consult the results of Makovitzki et al. [37] who investigated synthetic lipo-tetrapeptides linked to C-12, C-14 and C-16 fatty acids. They observed varying anti-fungal, anti-bacterial and hemolytic activities depending on the respective peptide sequence and length of the fatty acid chain. Their effect in vivo corresponded with the respective lipopeptide’s ability to disrupt the membrane of the respective organisms, indicating a membranolytic mode of action. Taking that together, **1** rather seemed to be a candidate for membranolytic bioactivity. Its anti-bacterial effect varies between the species and already between the two Gram positive bacteria MRSA and *S. agalactiae*. Taking all together, we conclude that **1** is not lysing the cells or affecting their integrity immediately. Taking the general bioactivity of lipopeptides into account, mostly affecting the cell membrane, possibly the lipid is interfering with the membrane during cell division, representing a more fragile state of cell integrity. It would be valuable to investigate its effect on melanoma cells more in detail, which was not possible in the present study due to a limited quantity of **1**.

After isolating **1**, the extracts of the bacterial fermentations were investigated upon the presence of the related Lipid 654 (**2**). The UPLC-MS/MS profiles of the solid phase extracts have not shown any signal that indicated the presence of **2**. However, it was reported that **2** is soluble in chloroform. To exclude that the lack of **2** was caused by unsuitability of solid phase extraction for that compound, we used chloroform liquid–liquid phase extraction and UHPLC-HR-MS to investigate its presence with negative outcome.

The natural role of *Algibacter*, being decomposers degrading algal biomass, may suggests that **1** is produced as a surfactant for mobilizing nutrients, in a similar way as the rhamnolipids do [38,39]. An additional role, or side effect, as an antibacterial agent cannot be excluded. There is no indication that the water insoluble Compound **2** is produced by the two *Algibacter* strains under the selected conditions; this could support the hypothesis that **1** is produced as a surfactant to mobilize hydrophobic nutrients. It furthermore supports the hypothesis that **1** is the biosynthetic precursor of **2** [17].

## 4. Materials and Methods

### 4.1. Bacterial Isolates

Two *Algibacter* sp. strains were isolated from organisms collected in the Barents Sea. Strain M09B557 was isolated from *Alcyonidium gelatinosum* sampled at 28.05.2009 at 70°6,60000’ N and 28°56,206190’ E. Strain M09B045 was isolated from *Gersemia rubiformis* sampled at the 14.05.2019 at 78°7,80000’ N and 13°34,962001’ E. The bacteria were isolated from the surface of the animals after washing them under filtrated seawater. Using a inoculation loop the surface of the organisms was sampled and potentially adhering bacteria were streaked out on FMAP agar, prepared of: 15 g Difco marine broth (Becton, Dickinson and Company, Franklin Lakes, NJ, USA), 15 g agar (Sigma, St. Louis, Mo, USA), 5 g peptone (Sigma), 700 mL ddH_2_O and 300 mL filtrated seawater. For storage of the isolates, liquid FMAP media was inoculated with the respective strain, grown until turbidity of the media was visible and cryo-conserved at −80 °C after adding 30% (*v*/*v*) glycerol (Sigma).

### 4.2. PCR and Identification of the Strains

The cryo-conserved isolates were plated out on FMAP agar in petri dishes and cultivated at 10 °C. After 7 d, colonies were picked and dissolved with 100 µL ddH_2_O in an Eppendorf tube. The sample was subsequently boiled for 5 min to break up the cells. For PCR, 1 µL of the bacterial lysate was used for a PCR reaction of 25 µL with 1 µM of forward and reverse primer (forward primer: 27F, AGAGTTTGATCMTGGCTCAG; reverse primer: 1492rR, CGGTTACCTTGTTACGACTT) and 12,5 µL ThermoPrimeTM 2 × ReddyMix PCR master mix (ThermoFisher Scientific, Waltham, MA, USA). The reactions were amplified using an Mastercycler epgradient S (Eppendorf, Hamburg, Germany) with the following program: 95 °C initial denaturation for 5 min followed by 30 cycles of 94 °C for 30 s, 55 °C for 30 s and 72 °C for 1 min. Final extension was at 72 °C for 10 min. Afterwards the PCR reaction was analyzed for purity on a 1.0% agarose gel and the results were documented using a Syngene Bioimaging system. For purification of the 16S rRNA gene PCR amplificate the QIAquick PCR purification kit was used according to manufacturer’s instructions (QIAGEN, Hilden, Germany). The PCR product purified from the gel was sequenced at the University Hospital of North Norway (Tromsø, Norway) employing the two primers mentioned above. For sequence homology comparison the online Basic Local Alignment Search Tool (BLAST) was used (www.ncbi.nlm.nih.gov/BLAST). The strains were identified according to their phylogenetic interference.

### 4.3. Fermentation and Extraction of Algibacter Cultures

M09B557 was cultivated in 1 L Warburg flasks containing 450 mL modified DSGC medium for 7 d at 130 rpm and 10 °C. M10B738 was cultivated for 12 d under the same conditions in 1 L Warburg flasks containing 450 mL DVR1 medium. Modified DSGC medium was prepared of 1 L filtrated seawater, 4.0 g d-glucose (Sigma) and 3.0 g Peptone (from casein, enzymatic digest, Sigma). DVR1 medium was prepared from 0.5 L filtrated seawater, 0.5 L ddH_2_O, 6.7 g malt extract (Sigma), 11.1 g Peptone (from casein, enzymatic digest, Sigma) and 6.7 g yeast extract (Sigma). All media were autoclaved at 120 °C for 30 min. The filtrated seawater was prepared by filtrating seawater through a Millidisk^®^ 40 Cartridge with Durapore^®^ 0.22 µm filter membrane (Millipore, Burlington, MA, USA).

For extraction of metabolites, solid phase extraction using Diaion^®^HP-20 resin (13607, Supelco Analytica, Bellefonte, PA, USA) was executed. The resin had been activated by incubation in methanol for a minimum of 30 min and washed with ddH_2_O for 15 min. 40 g of resin were added to 1 L of culture three days before the culture was harvested. The resin was separated from the fermentation broth by vacuum filtration using a cheesecloth mesh (1057, Dansk Hjemmeproduktion, Ejstrupholm, Danmark) to restrain the resin. Thereafter the resin was washed with 100 mL of ddH_2_O to remove remaining fermentation broth. The molecules bound to the resin were eluted with 150 mL methanol (HiPerSolv, VWR, Radnor, Penns., USA) per 40 g resin (shaking at 130 rpm for 30 min) and vaccuumfiltration using Whatman No. 3 filterpaper (Whatman plc, Buckinghamshire, UK). The elution from the resin was done twice and the methanolic extract was dried under reduced pressure at 40 °C and stored at −20 °C upon further processing.

### 4.4. Fractionation

Crude extracts were fractionated using flash liquid chromatography. The extracts were loaded onto resin (Diaion^®^ HP-20ss, Supelco) by dissolving them in 90% methanol aq. (*v*/*v*) and adding resin in a ratio of 1:1.5 (resin/dry extract, *w*/*w*). Subsequently, the solution was dried under reduced pressure at 40 °C. Flash columns (Biotage^®^ SNAP Ultra, Biotage, Uppsala, Sweden) were prepared by activating the resin by incubation in methanol for 20 min, washing with ddH_2_O and loading it into the column ensuring the resin being always covered with water. 6.5 g HP-20ss resin was loaded on one column. The fractionation was performed using a Biotage SP4™ system and a water: methanol gradient from 5–100% methanol over 36 min (6 min 5% B, 6 min 25% B, 6 min 50% B, 6 min 75% B, 12 min 100% B) followed by a methanol: acetone step-gradient (4 min methanol, 12 min acetone). The flow rate was set to 12 mL/min. 27 eluent fractions to 24 mL each were collected in glass tubes and pooled to six flash fractions in total (1–3 were pooled to fraction 1; 4–6 to fraction 2; 7–9 to fraction 3; 10–12 to fraction 4; 13–15 to fraction 5; 16–27 to fraction 6). An appropriate amount of extract-resin mixture was loaded onto the column after equilibration to 5% methanol aq. (*v*/*v*). The flash fractions were dried under reduced pressure at 40 °C.

### 4.5. UHPLC-HR-MS and Dereplication

UHPLC-HR-MS data for dereplication was recorded using an Acquity I-class UPLC (Waters, Milford, MA, USA) coupled to a PDA detector and a Vion IMS QToF (Waters). The chromatographic separation was performed using an Acquity C-18 UPLC column (1.7 µm, 2.1 mm × 100 mm) (Waters). Mobile phases consisted out of acetonitrile (HiPerSolv, VWR) for mobile phase A and ddH_2_O produced by the in-house Milli-Q system as mobile phase B, both containing 1% formic acid (*v*/*v*) (33015, Sigma). The gradient was run from 10% to 90% B in 12 min at a flow rate of 0.45 mL/min. Samples were run in ESI+ and ESI- ionization mode. The data was processed and analyzed using UNIFI 1.8.2 (Waters).

### 4.6. Isolation of Lipid 430

Purification of compound **1** was done using a semi preparative HPLC system (Waters) made up by a 600 HPLC pump, a 3100 mass spectrometer, a 2996 photo diode array detector and a 2767 sample manager. A 515 HPLC pump and a flow splitter were used to infuse the analytes into the MS. The mobile phases were degassed by an in-line degasser. For controlling the system, the software MassLynx™ 4.1 (Waters) was used. The columns used for isolation were X-Terra RP-18 preparative column (10 µm, 10 mm × 250 mm) and XSelect CSH preparative Fluoro-Phenyl column (5 µm, 10 mm × 250mm), both columns were purchased from Waters. The mobile phases for the gradients were A [ddH_2_O with 0.1% (*v*/*v*) formic acid] and B [acetonitrile with 0.1% (*v*/*v*) formic acid], flow rate was set to 6 mL/min. Acetonitrile (Prepsolv^®^, Merk KGaA, Darmsatdt, Germany) and formic acid (33015, Sigma) were purchased in appropriate quality, ddH_2_O was produced with the in-house Milli-Q^®^ system. For the MS-detection of the eluting compounds one percent of the flow was split from the fractions in line, blended with 80% Methanol in ddH_2_O (*v*/*v*) acidified with 0.2% Formic acid (Sigma) and directed to the ESI-quadrupole-MS.

### 4.7. NMR Spectroscopy

All NMR spectra were recorded on a Bruker Avance III HD spectrometer equipped with an inverse detected TCI probe with cryogenic enhancement on ^1^H, ^2^H and ^13^C. The operating frequencies were 599.90 MHz for ^1^H and 150.86 MHz for ^13^C. The samples were prepared in methanol-*d*_3_ and recorded at 298 K.

All experiments were recorded using standard pulse sequences for Proton, Presat, Carbon, DQFCOSY, HSQC, HMBC, H2BC, NOESY and ROESY (gradient selected and adiabatic versions, with matched sweeps where applicable) in Topspin 3.5pl7 and processed in Mnova 12.0.0. Spectra were referenced on the residual solvent peak of methanol-*d*_3_ (δH = 3.31 and δC = 49.00).

### 4.8. Lipid Extraction

Total lipids were extracted by shaking 25 mL of bacterial culture with 25 mL chloroform (EMSURE^®^, Merck) in screw cap centrifuge tubes (21008-242, VWR) for 3 h at 40 rpm using a tube-rotator (SB3, Stuart, Stone, UK). Afterwards the organic phase was separated and centrifuged for 10 min at 4600 rpm (Multifuge 3, rotor 75006445, S-R, Heraeus, Hanau, Germany) to remove debris and particles. Thereafter the organic phase was vacuum filtrated trough Whatman No. 3 filter paper (Whatman) and concentrated to 5 mL under nitrogen. 

### 4.9. Anti-Microbial Assays

#### 4.9.1. Growth Inhibition Assay 

To determine and quantify anti-microbial activity, a bacteria growth inhibition assay in liquid media was executed. The samples were tested against *S. aureus* (ATCC 25923), *E. coli* (ATCC 259233), *E. faecialis* (ATCC 29122), *P. aeruginosa* (ATCC 27853), *S. agalactiae* (ATCC 12386) and Methicillin resistant *S. aureus* (MRSA) (ATCC 33591). *S. aureus*, MRSA, *E. coli* and *P. aeruginosa* were grown in Muller Hinton broth (275730, Becton, Dickinson and Company). *E. facalis* and *S. agalactiae* were cultured in brain hearth infusion broth (53286, Sigma). Fresh bacteria colonies were transferred in the respective medium and incubated at 37 °C over night. The bacterial cultures were diluted to a culture density representing the log phase and 50 µL/well were pipetted into a 96-well microtiter plate (734-2097, Nunclon™, Thermo Scientific, Waltham, MA, USA). The final cell density was 1500–15,000 CFU/well. Flash fractionated extracts were diluted in 1% (*v*/*v*) dimethyl sulfoxide (DMSO, D4540, Sigma). Pure compound was diluted in 2% (*v*/*v*) DMSO in ddH_2_O, the final assay concentration was 50% of the prepared sample, since 50 µL of sample in DMSO/water were added to 50 µL bacterial culture. After adding the samples to the plates, they were incubated over night at 37 °C and the growth was determined by measuring the optical density at λ = 600 nm (OD_600_) with a 1420 Multilabel Counter VICTOR^3^™ (Perkin Elmer, Waltham, MA, USA). A water sample was used as reference control, growth medium without bacteria was used as a negative control and a dilution series of gentamycin (A2712, Merck) from 32 to 0.01 µg/mL was used as positive control and visually inspected for bacterial growth. The positive control was used as system suitability test and the results of the antimicrobial assay were only considered valid when positive control was passed. The final concentration of DMSO in the assays was ≤ 2% (*v*/*v*) known to have no effect in the tested bacteria. The data was processed using GraphPad Prism 8.

#### 4.9.2. Bactericidal Assay

For investigation of bactericidal effect, the 100 µL reactions of the *S. agalactiae* anti-microbial assay were streaked on brown agar plates (University hospital of northern Norway, Tromsø, Norway) and incubated for four days at 37 °C, the plates were visually investigated after 1 day and 4 days of incubation.

### 4.10. Cell Proliferation Assay

The inhibitory effect of fractions and compounds was tested using an MTS in vitro cell proliferation assay against two cancer cell lines and one normal cell line. The cancer cell lines were human melanoma A2058 (ATCC, CLR-1147™) and human colon carcinoma HT29 (ATCC HTB-22™), as cell line for the general cytotoxicity assessment, non-malignant MRC5 lung fibroblast cells (ATCC CCL-171™) were employed. The cells were cultured and assayed in Roswell Park Memorial Institute medium (RPMI-16040, FG1383, Merck) containing 10% (*v*/*v*) Fetal Bovine serum (FBS, 50115, Biochrom, Cambridge, UK). The cell-concentration was 4000 cells/well for the lung fibroblast cells and 2000 cells/well for the cancer cells. After seeding, the cells were incubated 24 h at 37 °C and 5% CO_2_. The medium was then replaced with fresh RPMI-1640 medium supplemented with 10% (*v*/*v*) FBS and gentamycin (10 µg/mL, A2712, Merck). After adding 10 µL of sample diluted in 2% (*v*/*v*) DMSO in ddH_2_O the cells were incubated for 72 h at 37 °C and 5% CO_2_. For assaying the viability of the cells 10 µL of CellTiter 96^®^AQ_ueous_ One Solution Reagent (G3581, Promega, Madison, WI, USA) containing tetrazolium [3-(4,5-dimethylthiazol-2-yl)-5-(3-carboxymethoxyphenyl)-2-(4-sulfophenyl)-2H-tetrazolium, inner salt] and phenazine ethosulfate was added to each well and incubated for one hour. The tests were executed with three technical replicates. The plates were read using a DTX 880 plate reader by measuring the absorbance at λ = 485 nm. The cell viability was calculated using the media control. As a negative control RPMI-1640 with 10% (*v*/*v*) FBS and 0.5% Triton™ X-100 (Sigma-Aldrich) was used as a positive control. The data was processed and visualized using GraphPad Prism 8

### 4.11. Mode of Action Studies 

#### 4.11.1. Flow Cytometry

For the investigation of the mode of action of **1** on the A2058 cells, cells were seed in six well plates (Nunclon™, Thermo Fisher) with a density of one million cells in three mL of Eagle’s medium (Dulbecco’s modified Eagles medium, D6171, Sigma) with 10% (*v*/*v*) FBS. Cells were incubated over night at 37 °C and 5% CO_2_ and medium was exchanged to two mL Eagle’s medium, the respective amount of compound or Triton™ X-100 (Sigma-Aldrich) as positive control, propidium iodide (Sigma) to a final concentration of 5 µg/mL and the wells were filled up to 3 mL with phosphate buffered saline (PBS, Dulbecco’s PBS, D8537, Sigma). One unstained control without propidium iodide and one negative control without compound were prepared. The reactions were incubated for 1 h at 37 °C and 5% CO_2_, media was removed, the cells were spilled with PBS buffer and trypsinated using 400 µL of trypsine solution (Trypsin-EDTA 10 ×, Biowest, Nuaillé, France) and redissolved in 1 mL of PBS with propidium iodide (5 µg/mL). The cell suspensions were transferred into sample tubes and analyzed using a Cytoflex flow cytometer (Beckman Coulter, Brea, Cal., US) and CytExpert.

#### 4.11.2. Microscopic Investigation of Melanoma Cells

For the microscopic investigation of melanoma cells, the A2058 cells were seed out in Eagle’s medium at a concentration of 2000 cells/well in 100 µL medium per well. The cells were incubated over night at 37 °C and 5% CO_2_. The media in the wells was replaced with 50 µL Eagle’s medium, compound **1** was added and the wells were filled up with PBS to a total volume of 100 µL. The reactions were incubated at 37 °C and 5% CO_2_ for 4 h, 50 µL of media were removed and replaced with 50 µL of 0.4% Trypan blue solution (Sigma) and incubated at room temperature for three minutes. Then the media with Trypan blue was removed, leaving the bottom of the wells covered with a thin liquid layer and examined microscopically at a magnification of 10 × 10 (Leica DMIC, Leica, Germany). Pictures were taken using a microscope camera (Marlin F-046B IRF, Allied vision, Germany).

## 5. Conclusions

It was shown that two strains of the genus *Algibacter* were capable of producing Lipid 430 (**1**). The bioactivity of **1** seems to be comparable to other lipopeptides such as synthetic lipo-tetrapeptides. It showed cytotoxicity against melanoma cells with a IC_50_ concentration of 175 µM after 72 h of incubation, the exact mode of action remains to be investigated but our experimental results indicate that **1** did not lyse the cells immediately. The IC_50_ concentration against *S. agalactiae* was determined to be 30 µM, at a concentration of 58 µM it was shown to be bactericidal against *S. agalactiae*. To the best of our knowledge, this is the first report of bioactive compounds isolated from the genus *Algibacter*.

## Figures and Tables

**Table 1:** ^1^H and ^13^C assignments for “Lipid 430 (**1**)” (see Figure 3) (^1^H 600 MHz, ^13^C 150 MHz, CD_3_OH).

	Lipid 430 (1)	
Position	δ_C_, Type	δ_H_ (*J* in Hz)
1	173.8, C	
2	56.5, CH	4.47, dt (8.2, 4.2)
3a	63.2, CH_2_	3.89, dd (9.3, 5.2) ) ^c^
3b		3.82, dd (11.3, 3.9)
4		7.99, d (7.8)
5	171.5, C	
6a	43.6, CH_2_	3.98, dd (16.7, 5.9) ^b^
6b		3.89, dd (9.3, 5.2) ^c^
7		8.31, t (5.9)
8	175.0, C	
9a	44.8, CH_2_	2.40, dd (13.9, 4.1)
9b		2.33, dd (14.0, 8.8)
10	70.0, CH	3.98, dd (16.7, 5.9) ^b^
11	38.3, CH_2_	1.50–1.47, m
12	26.5, CH_2_	1.47–1.42, m
13	30.9–30.6 ^a^, CH_2_	1.29, p (6.1, 5.4) ^e^
14	30.9–30.6 ^a^, CH_2_	1.29, p (6.1, 5.4) ^e^
15	30.9–30.6 ^a^, CH_2_	1.29, p (6.1, 5.4) ^e^
16	30.9–30.6 ^a^, CH_2_	1.29, p (6.1, 5.4) ^e^
17	30.9–30.6 ^a^, CH_2_	1.29, p (6.1, 5.4) ^e^
18	30.9–30.6 ^a^, CH_2_	1.29, p (6.1, 5.4) ^e^
19	30.9–30.6 ^a^, CH_2_	1.29, p (6.1, 5.4) ^e^
20	28.4, CH_2_	1.29, p (6.1, 5.4) ^e^
21	40.1, CH_2_	1.16, q (7.1, 6.7)
22	29.0, CH	1.56–1.50, m
23	22.9, CH_3_	0.87, d (6.6) ^d^
24	22.9, CH_3_	0.87, d (6.6) ^d^

^a–e^ Signals are overlapping.

## Supplementary Materials

The following are available online, Figure S1: purity analysis of prepared compound **1**, Figure S2: ^1^H NMR spectrum of **1**, Figure S3: ^13^C NMR spectrum of **1**, Figure S4: HSQC + HMBC spectrum of **1**, Figure S5: COSY spectrum of **1**, Figure S6: H2BC spectrum of **1**, Figure S7: results of the cytotoxicity assays, Figure S8: results of the flow cytometry experiments, Figure S9: pictures of the microscopic investigation of melanoma cells.

## Appendix A

16S rRNA sequencing results

**Strain ID**	**Genus**	**16s rRNA sequence**
M09B557	*Algibacter* sp.	TTGGGTTTAAGGGTCCGTAGGTGGATAATTAAGTCAGAGGTGAAAGTTTGCAGCTCAACTGTAAAATTGCCTTTGATACTGGTTATCTTGAATCATTATGAAGTGGTTAGAATATGTAGTGTAGCGGTGAAATGCATAGATATTACATAGAATACCAATTGCGAAGGCAGATCACTAATAATGTATTGACACTGATGGACGAAAGCGTGGGGAGCGAACAGGATTAGATACCCTGGTAGTCCACGCCGTAAACGATGGATACTAGCTGTTCGGAACTTGTTTCTGAGTGGCTAAGCGAAAGTGATAAGTATCCCACCTGGGGAGTACGTTCGCAAGAATGAAACTCAAAGGAATTGACGGGGGCCCGCACAAGCGGTGGAGCATGTGGTTTAATTCGATGATACGCGAGGAACCTTACCAGGGCTTAAATGTAGATTGACAGGACTAGAGATAGTTTTTTCTTCGGACAATTTACAAGGTGCTGCATGGTTGTCGTCAGCTCGTGCCGTGAGGTGTCAGGTTAAGTCCTATAACGAGCGCAACCCCTGTTGTTAGTTGCCAGCGAGTCAAGTCGGGAACTCTAACAAGACTGCCAGTGCAAACTGTGAGGAAGGTGGGGATGACGTCAAATCATCACGGCCCTTACGTCCTGGGCTACACACGTGCTACAATGGTAGGGACAGAGAGCAGCCACTGGGCGACCAGGAGCGAATCTATAAACCCTATCACAGTTCGGATCGGAGTCTGCAACTCGACTCCGTGAAGCTGGAATCGCTAGTAATCGCATATCAGCCATGATGCGGNGAATACGTTCCCGGGNNNT
M09B045	*Algibacter* sp.	TGANNGTTTGCAGCTCANNNNNNAAATTGCCTTTGATACNNGTTATCTTGAATCATTATGANNNNNNTAGANTNNGNANNNNNGCGGTGAAATGCATAGATATTACATAGAATACCAATTGCGAAGGCAGATCACTAATAATGTATTGACACTGATGGACGAAAGCGTGGGGAGCGAACAGGATTAGATACCCTGGTAGTCCACGCCGTAAACGATGGATACTAGCTGTTCGGAACTTGTTTCTGAGTGGCTAAGCGAAAGTGATAAGTATCCCACCTGGGGAGTACGTTCGCAAGAATGAAACTCAAAGGAATTGACGGGGGCCCGCACAAGCGGTGGAGCATGTGGTTTAATTCGATGATACGCGAGGAACCTTACCAGGGCTTAAATGTAGATTGACAGGACTAGAGATAGTTTTTTCTTCGGACAATTTACAAGGTGCTGCATGGTTGTCGTCAGCTCGTGCCGTGAGGTGTCAGGTTAAGTCCTATAACGAGCGCAACCCCTGTTGTTAGTTGCCAGCGAGTCATGTCGGGAACTCTAACAAGACTGCCAGTGCAAACTGTGAGGAAGGGGGGGGGATGACGTCAAATCATCACGGCCCTTACGTCCTGGGCTACACACGTGCTACAATGGTAGGGACAGAGAGCAGCCACTGGGCGACCAGGAGCGAATCTATAAACCCTATCACAGTTCGGATCGGAGTCTGCAACTCGACTCCGTGAAGCTGGAATCGCTAGTAATCGCATATCAGCCATGATGCGGTGAATACGTTCCCGGGCCTTGTACACACCGCCCGTCAAGCCATGGAAGCTGGGANTGNCTGAAGTCCGTCACCGTAAGGGAGCGGGC
