# Supplementary material for: Anti-Bacterial Effect and Cytotoxicity Assessment of Lipid 430 Isolated from Algibacter sp
Source: Molecules. 2019 Nov 5;24(21):3991. doi: 10.3390/molecules24213991 (PMC6864645; doi:10.3390/molecules24213991)
Supplement: Supplementary file 1 [file molecules-24-03991-s001.pdf]

Supplementary Information

Purity of the prepared Lipid 430 (1) by UHPLC-DAD-MS/MS

Figure S1 ESI− BPI chromatogram (A), Extracted Ion chromatogram of **1** (B) and $A_{254\ nm}$ chromatogram (C)

NMR Spectroscopic Data for Lipid 430 (1)

Figure S2 ^{1}H NMR (600 MHz, CD_3OD) spectrum of **1**

Figure S3 ^{13}C NMR (151 MHz, CD_3OD) spectrum of **1**

Figure S4 HSQC + HMBC (600 MHz, CD_3OD) spectrum of **1**

Figure S5 COSY (600 MHz, CD_3OD) spectrum of **1**

Figure S6 H2BC (600 MHz, CD_3OD) spectrum of **1**

Results of the cytotoxicity assay

Figure S7 Results of the cytotoxicity assays for all tested cell lines

Results of the mode of action studies for all concentrations of Lipid 430 (1) and controls

Figure S8 Results of the flow cytometry experiments with propidium iodide staining

Figure S9 Pictures of the microscopic investigation

Figure S1. ESI− BPI chromatogram (A), Extracted Ion chromatogram of Lipid 430 (B) and $A_{254\ nm}$ (C)

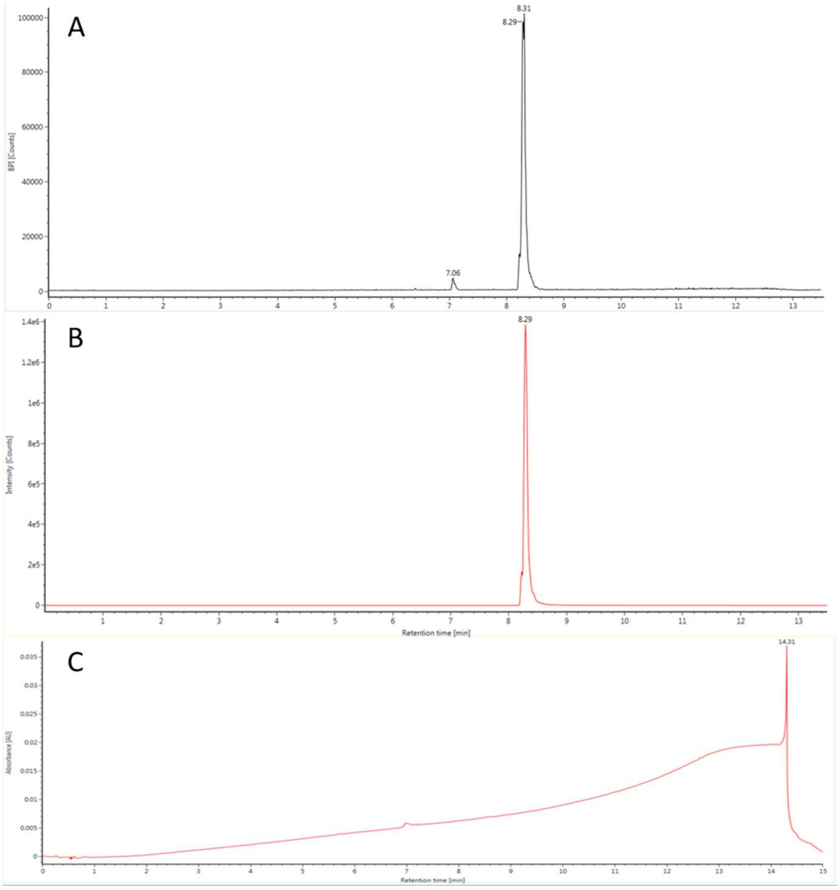

Chromatograms of the UHPLC analysis of the isolated Lipid 430 (**1**). In A the base peak intensity chromatogram of the ESI-MS/MS signal is depicted. In B the extracted ion chromatogram of the most abundant isotopic peak (m/z 429.2972, $[M\text{-}H]^-$) and in C the absorption at 254 nm. The signal at RT = 7.06 min is also visible when injecting the blank solution.

Figure S2. ^{1}H NMR (600 MHz, CD_3OD) spectrum of **1**

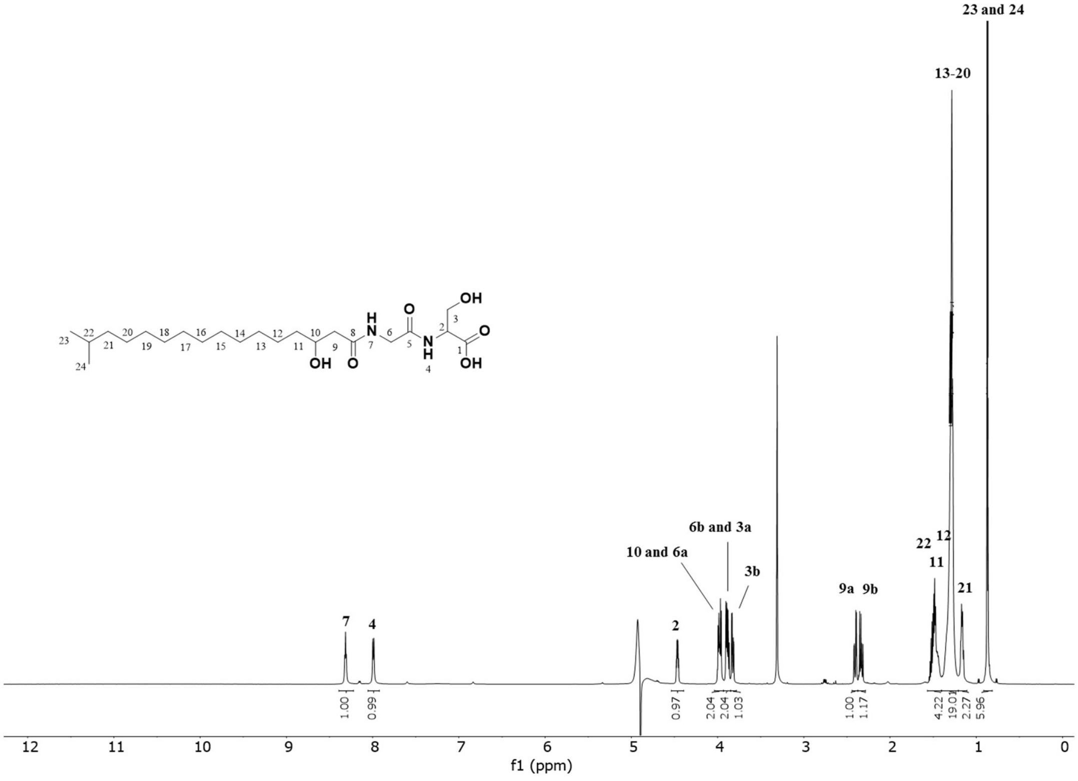

Figure S3. ^{13}C NMR (151 MHz, CD_3OD) spectrum of **1**

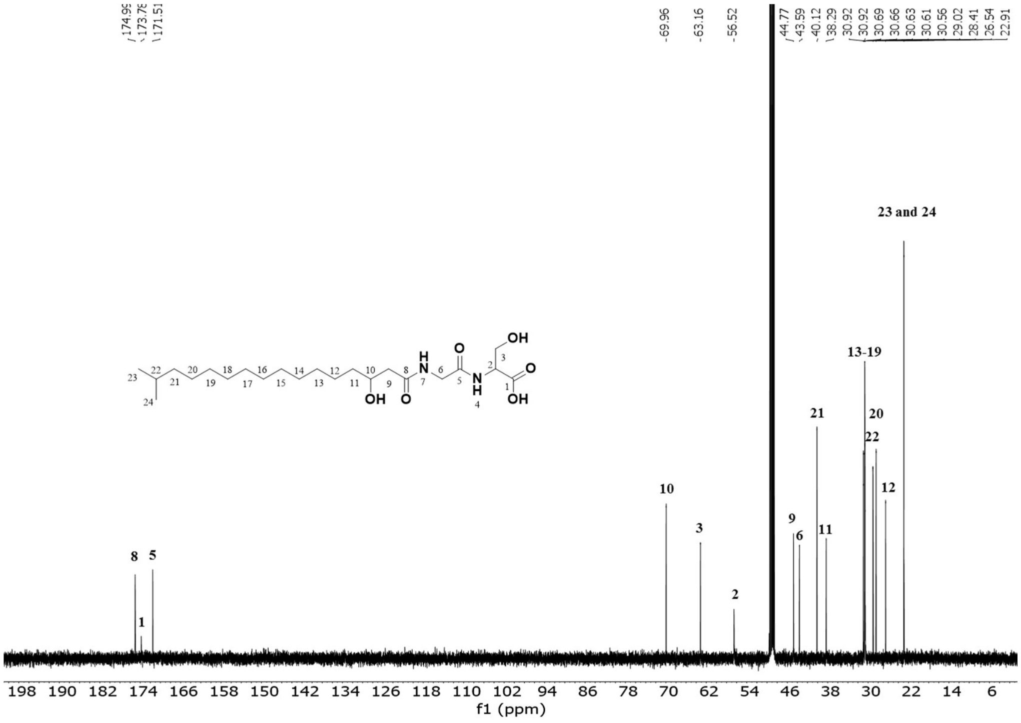

Figure S4. HSQC + HMBC (600 MHz, CD_3OD) spectrum of **1**

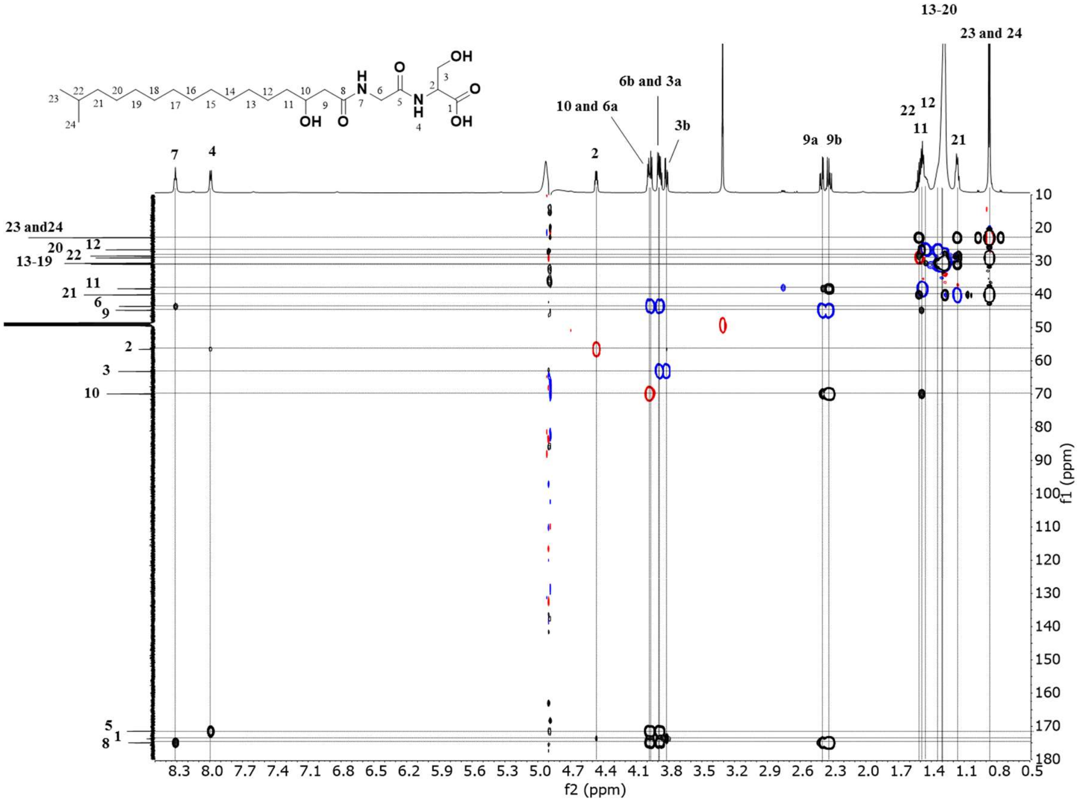

Figure S5. COSY (600 MHz, CD_3OD) spectrum of **1**

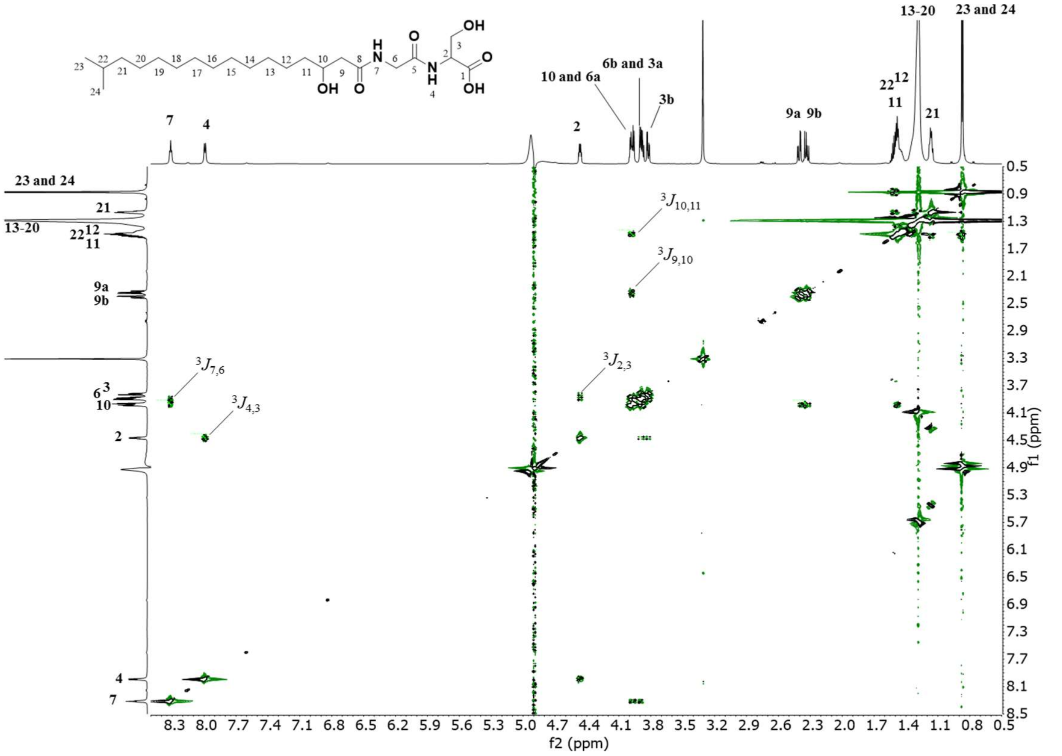

Figure S6. H2BC (600 MHz, CD_3OD) spectrum of **1**

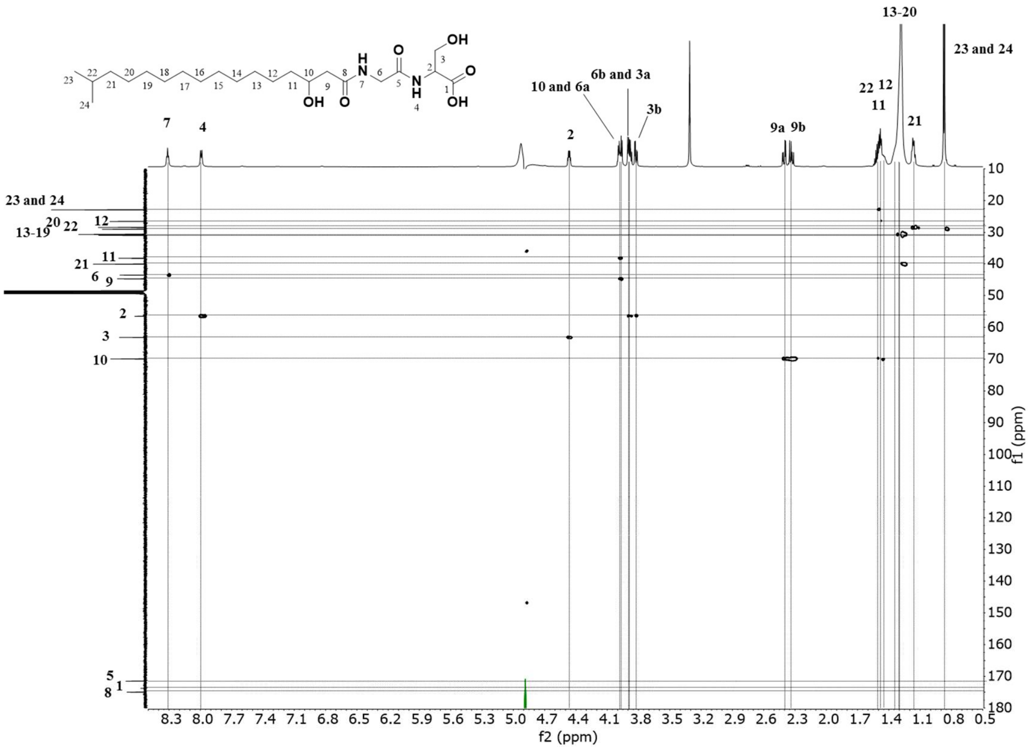

Figure S7. Results of the cytotoxicity assays for all tested cell lines

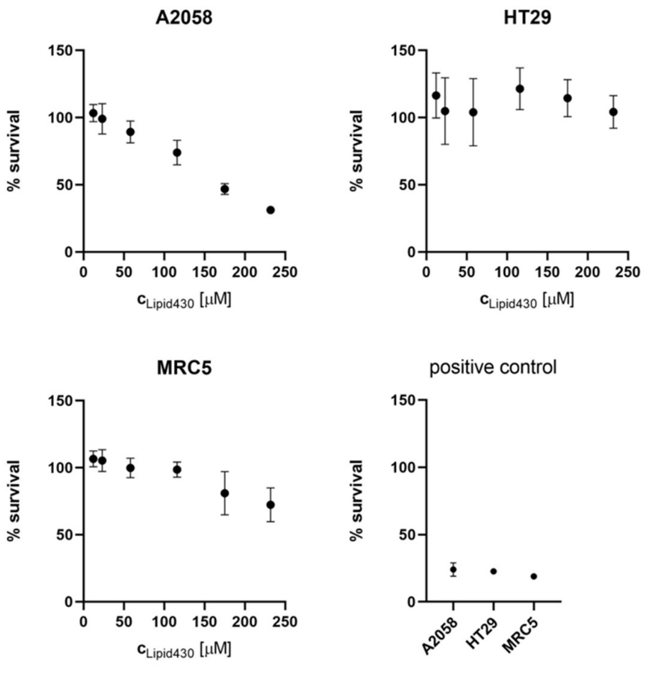

Cytotoxicity assay results for the melanoma (A2058), colon carcinoma (HT29) and lung fibroblast (MRC5) cells. The assay result is given as % survival on the y-axis and the concentrations of Lipid 430 on the x-axis. The exact tested concentrations were of 233, 175, 116, 58, 23 and 12 μM or 100, 75, 50, 25, 10 and 5 μg/mL respectively. 0.5% Triton™ X-100 was used as positive control.

Figure S8. Results of the flow cytometry experiments with propidium iodide staining

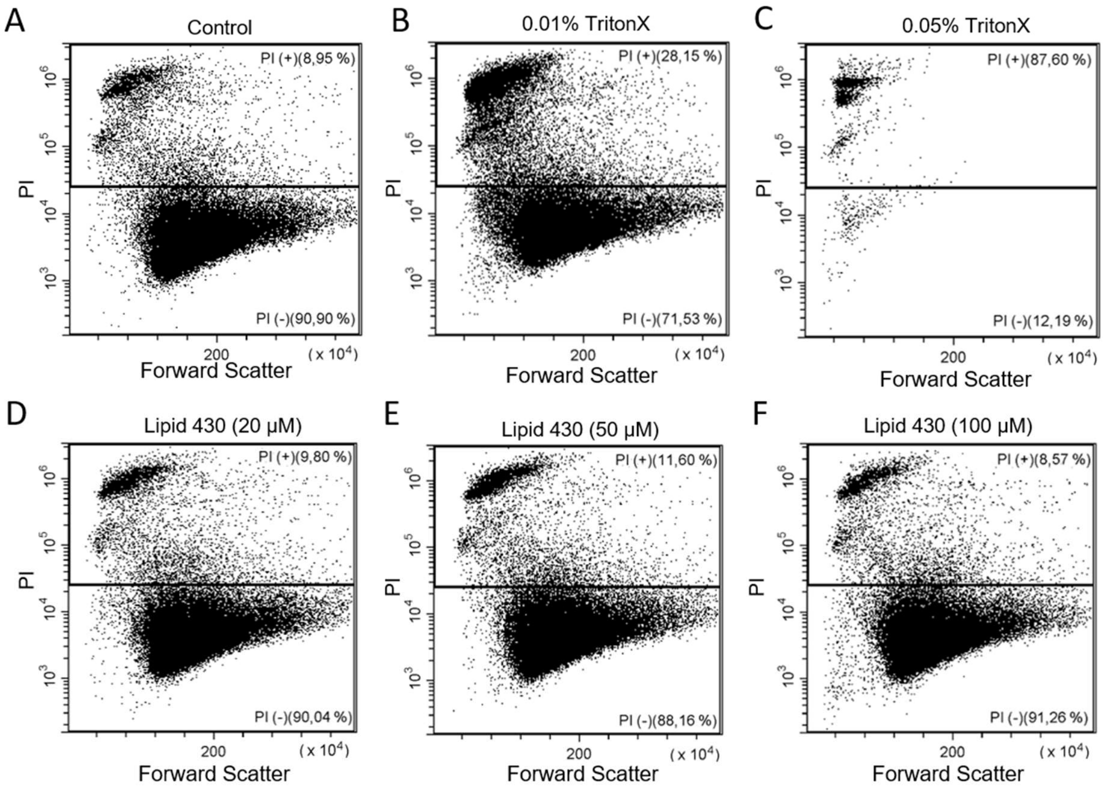

DotPlot graphs of the flow cytometry experiments with melanoma cell line A2058. In the upper sections the propidiumiodide positive (PI+) events (cell integrity destroyed/ affected) and in the lower the propidiumiodide negative events (PI -, physiologic cells). Forward scatter is displayed on the x-axis and propidiumiodide absorption on the y-axis. The relative ratio of events is given in %. A: stained control, 8.95% PI+; B: 0.01% TritonX, 28.15% PI+; C: 0.05% TritonX, 87.60% PI+; D: 20 µM Lipid 430, 9.80% PI+; E: 50 µM Lipid 430, 11.60% PI+; F: 100 µM Lipid 430, 8.57% PI+.

Figure S9. Results of the microscopic investigation

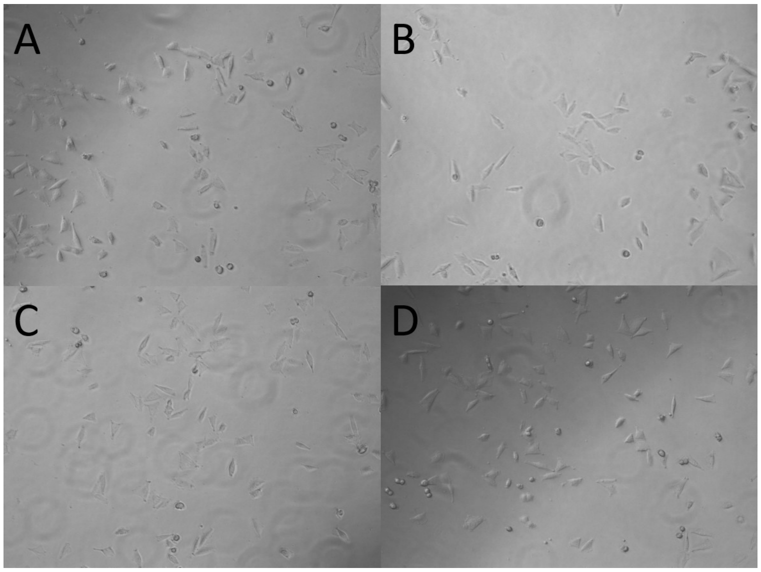

Microscopic investigation of Melanoma cells (A2058) after 1h of incubation with test solution. Inspection was done at 100× magnification. A: PBS-control; B: 1% (v/v) DMSO; C: Lipid 430, 100 µg/mL; D: Lipid 430, 500 µg/mL.
